# Comprehensive analysis of lumbar disc degeneration and autophagy-related candidate genes, pathways, and targeting drugs

**DOI:** 10.1186/s13018-021-02417-2

**Published:** 2021-04-13

**Authors:** Wei-long Xu, Yan Zhao

**Affiliations:** 1 Inner Mongolia Medical University, Hohhot, 010000 China; 2 Department of Thoracolumbar Spine Surgery, The Second Affiliated Hospital of Inner Mongolia Medical University, Hohhot, 010000 China

**Keywords:** Bioinformatics-based analysis, Targeting drug, Autophagy, Gene, Lumbar disc degeneration, Pathway

## Abstract

**Background:**

Lumbar disc degeneration (LDD) is an essential pathological mechanism related to low back pain. Current research on spinal surgery focused on the sophisticated mechanisms involved in LDD, and autophagy was regarded as an essential factor in the pathogenesis.

**Objectives:**

Our research aimed to apply a bioinformatics approach to select some candidate genes and signaling pathways in relationship with autophagy and LDD and to figure out potential agents targeting autophagy- and LDD-related genes.

**Materials and methods:**

Text mining was used to find autophagy- and LDD-related genes. The DAVID program was applied in Gene Ontology and pathway analysis after selecting these genes. Several important gene modules were obtained by establishing a network of protein-protein interaction and a functional enrichment analysis. Finally, the selected genes were searched in the drug database to find the agents that target LDD- and autophagy-related genes.

**Results:**

There were 72 genes related to “autophagy” and “LDD.” Three significant gene modules (22 genes) were selected by using gene enrichment analysis, which represented 4 signaling pathways targeted by 32 kinds of drugs approved by the Food and Drug Administration (FDA). The interactions between drugs and the genes were also identified.

**Conclusion:**

To conclude, a method was proposed in our research to find candidate genes, pathways, and drugs which were involved in autophagy and LDD. We discovered 22 genes, 4 pathways, and 32 potential agents, which provided a theoretical basis and new direction for clinical and basic research on LDD.

## Introduction

For low back pain, lumbar disc degeneration (LDD) is an essential pathological mechanism involved [1]. According to the previous studies, lumbar degenerative conditions including lumbar instability and stenosis as well as disc herniation lumbar stenosis often occur following LDD [2, 3]. Multiple factors may contribute to LDD, including nutrition, injury, spinal biomechanics, inflammation, and biology [4, 5]. Recent studies on spinal surgery mainly focused on the sophisticated mechanisms related to LDD [6], and autophagy was regarded as an essential pathological factor.

Autophagy is an intracellular catabolic process dependent on lysosome which involves the degradation of protein aggregates, organelles, and cytoplasmic proteins. Pathologically enhanced autophagy was considered to be related to cell death in certain scenarios; however, it plays a protective role in various circumstances [7].

In the past decade, multiple studies on gene expression profiling worked on autophagy and LDD, with piles of candidate genes identified [8, 9]. The purpose of our research was to find candidate genes and signaling pathways in relationship with autophagy and LDD by using a bioinformatics approach and to discover drugs that target LDD- and autophagy-related genes. Firstly, through text mining, we detected the genes associated with autophagy and LDD. Subsequently, the online bioinformatics resource DAVID was used to perform signaling pathway and functional analyses. Then, protein-protein interaction (PPI) networks were established by using the genes both related to autophagy and LDD, with 3 important gene modules identified. Finally, candidate drugs were identified according to the gene-drug interaction analysis by using the selected genes. Through this method, we determined some potential key genes, signaling pathways, and drugs, which provide new insights for clinical and basic research on LDD.

## Materials and methods

### Text mining

Text mining was conducted by using the GenCLip3 platform (http://ci.smu.edu.cn/genclip3/analysis.php). The names of all genes available in the published reports associated with the concept of the search were collected by using GenCLip3 [10]. LDD and autophagy were respectively searched, and the genes related to both autophagy and LDD were selected for further analysis.

### Gene Ontology (GO) enrichment and pathway analysis

In Gene Ontology enrichment analysis [11], gene products were depicted according to the following 3 parts: cellular component(CC), molecular function (MF), and biological process (BP). Data resources of available metabolic pathways were collected from the Kyoto Encyclopedia of Genes and Genomes (KEGG) [12]. Genes related to both terms were selected by using the DAVID [13] for further GO and KEGG enrichment. *P* <0.05 indicated statistical significance.

### PPI networks and module analysis

The Search Tool for the Retrieval of Interacting Genes (STRING, version 11.0) [14] database was applied to establish a network of protein-protein interaction of the genes related to both autophagy and LDD. The significant threshold was set as interaction value >0.900 (the highest reliability). PPI networks were then established by applying the Cytoscape software [15]. The molecular complex detection (MCODE) is an automatic approach for analyzing highly associated modules as molecular clusters or complexes.

### The interaction between drugs and genes

The Drug Gene Interaction Database (DGIdb, http://www.dgidb.org) was used to reveal the interactions between drugs and the selected genes [16]. Potential drugs targeting the genes related to autophagy and LDD could provide new insight into therapeutic strategies.

## Results

### Results of text mining

**Fig. 1:**
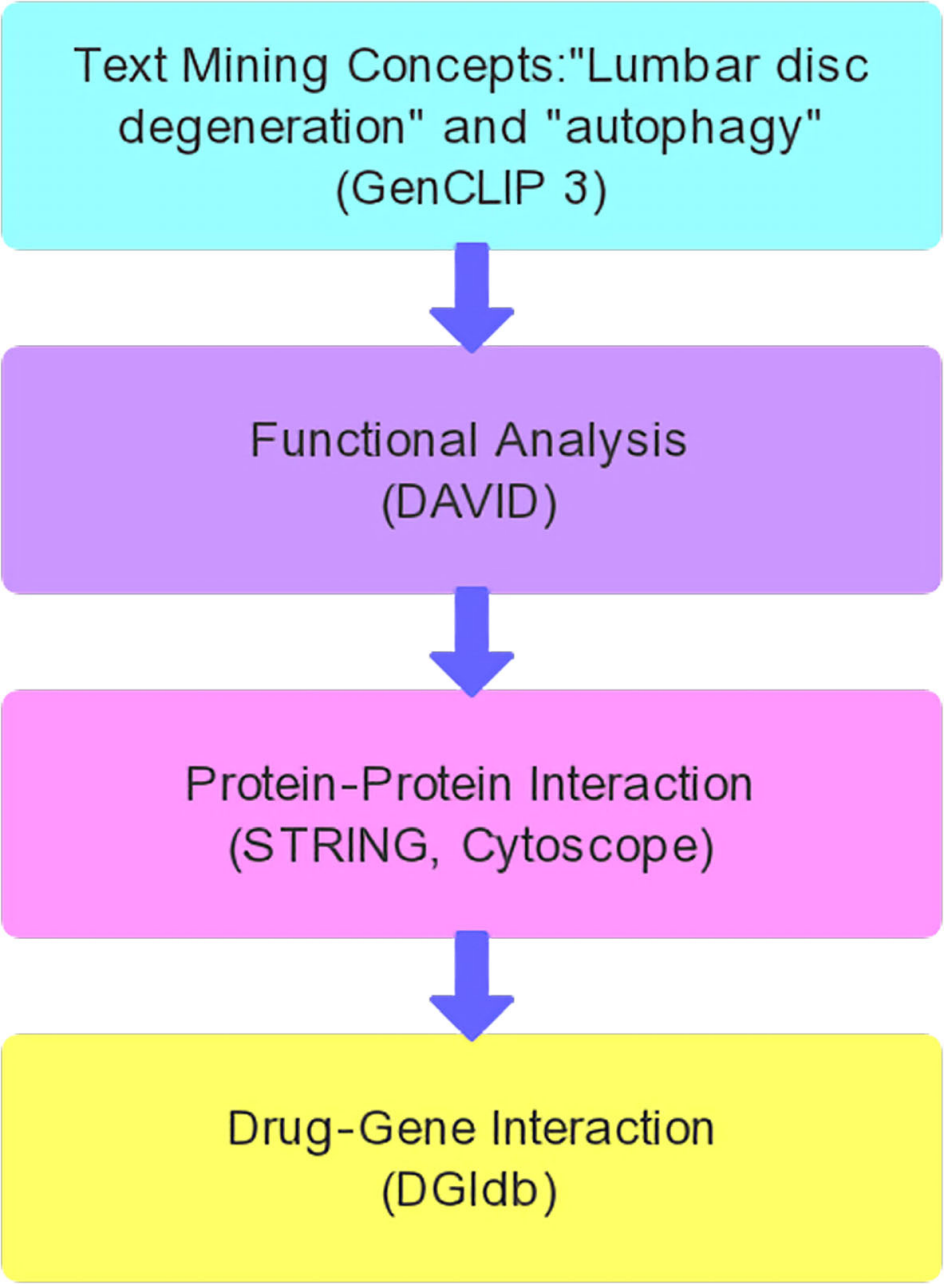
Overall data mining strategy

**Fig. 2:**
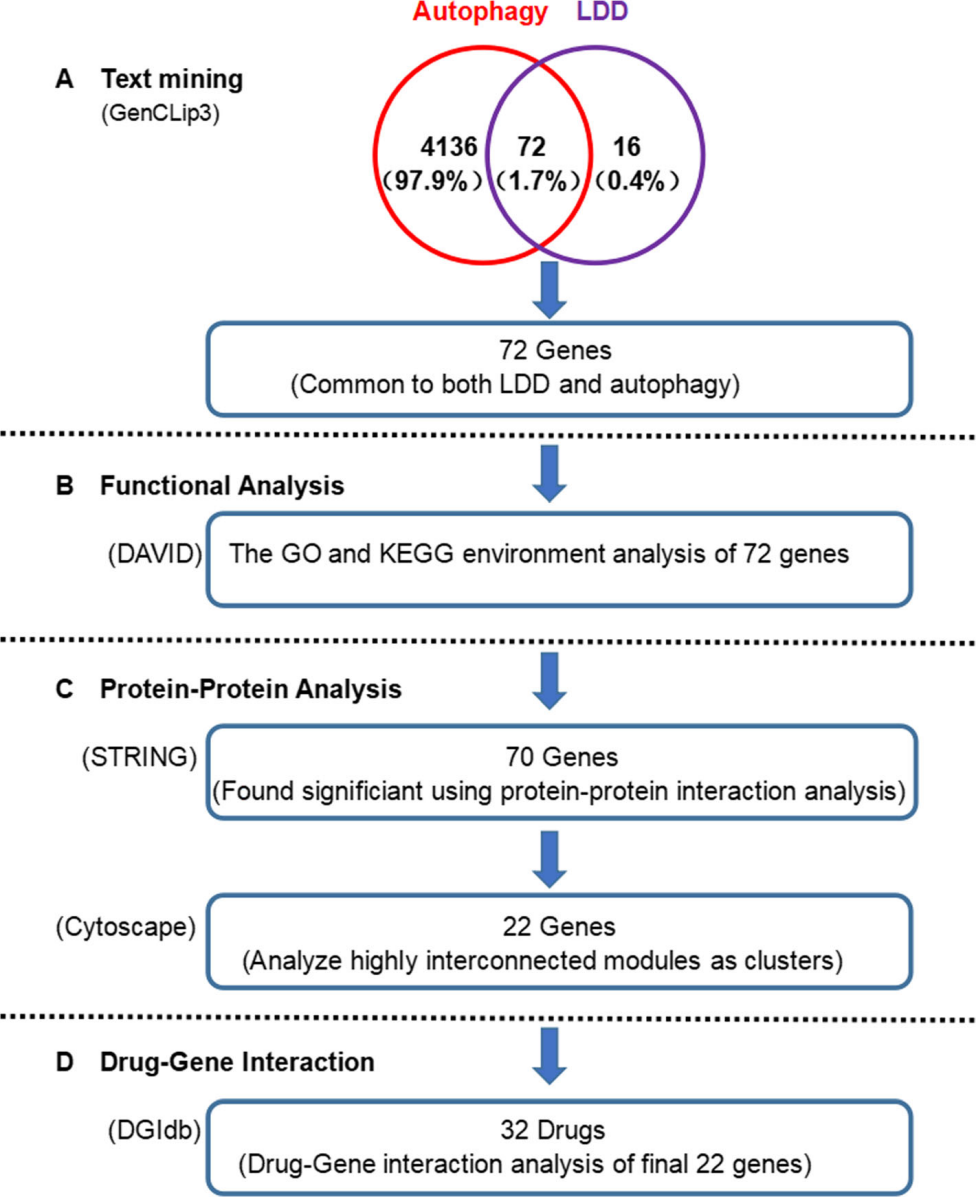
Summary of data mining results

According to the mining strategy shown in Fig. 1, 4208 genes were identified associated with autophagy and 88 genes with LDD. There were 72 genes related to both autophagy and LDD (Table 1 and Fig. 2).

**Table 1:** Seventy-two genes related to both autophagy and LDD were identified

ACAN	CASP9	FASLG	IL6	MSTN	TIMP1
ADAMTS5	CCL5	FGFR1	IL6ST	NOS2	TLR4
ADIPOQ	CDKN2A	FGFR3	LEP	NOS3	TNF
AKT1	COL1A1	GSR	LIF	PCBD1	TNFRSF10A
AQP3	COL2A1	HSPA8	MAPK1	PIK3CA	TNFRSF11B
BAX	CSF1	IGF1	MIR100	PPM1D	TNFSF10
BCL2	CSF1R	IGF1R	MIR146A	PRIMA1	TP53
BDNF	CTGF	IL10	MMP1	PTH	TRPC6
BGLAP	CX3CL1	IL1A	MMP13	PTK2B	TRPV4
BMPR2	CX3CR1	IL1B	MMP2	SOX9	TSLP
CALCA	CXCL12	IL1RN	MMP3	SPARC	VDR
CASP3	FAS	IL4	MMP9	STAT3	VEGFA

### Gene Ontology enrichment and pathway analysis

**Fig. 3:**
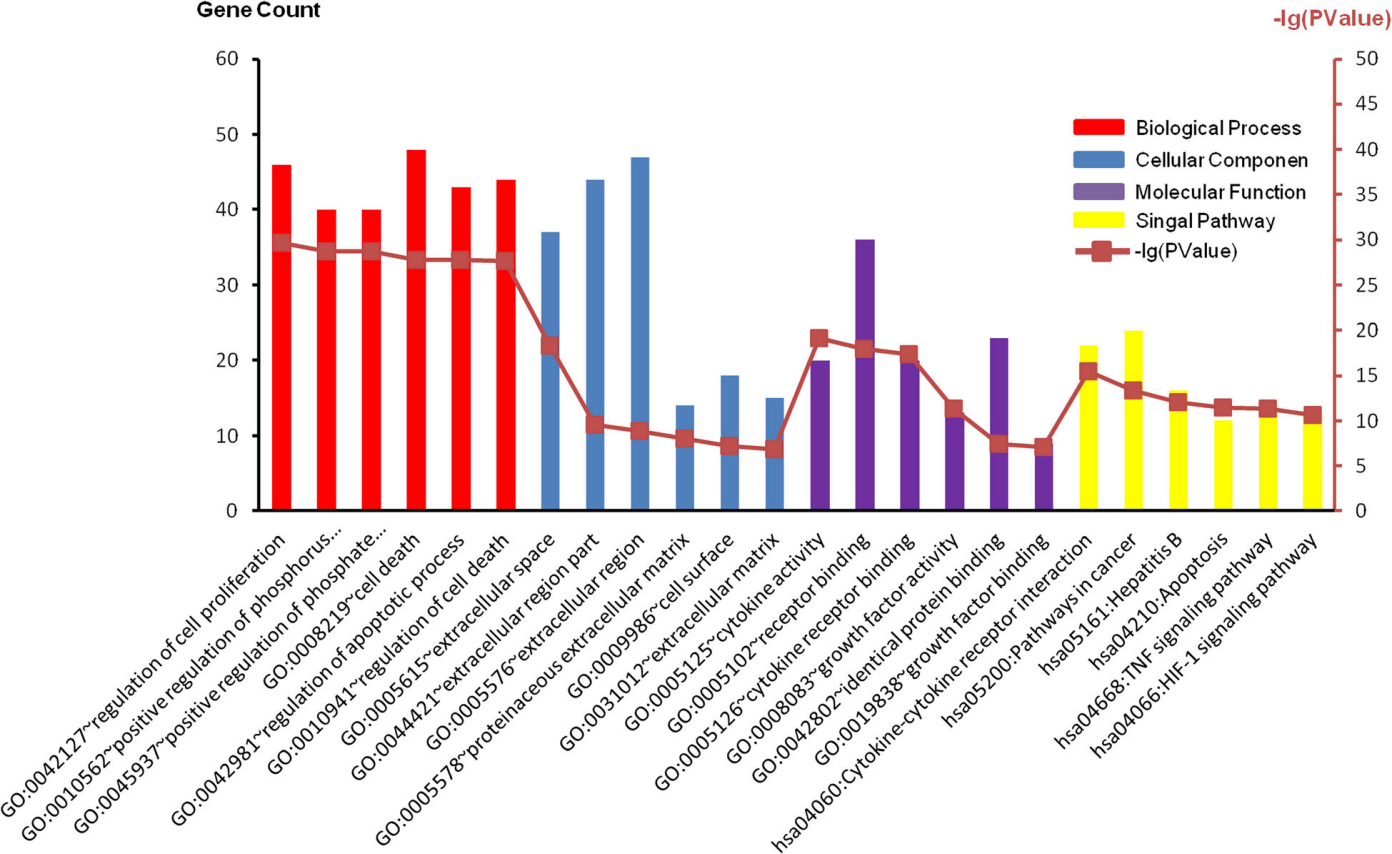
GO terms and KEGG pathways of the 72 genes related to both autophagy and LDD

GO and KEGG analyses were performed by using the 72 genes, and *P* value <0.05 was set as the standard (Fig. 3). In Fig. 3, the top 6 important terms were shown respectively.

Furthermore, the annotation of these genes is presented in Table 2. It was found that in the biological process group, the selected genes were mostly associated with cell proliferation, cellular death and apoptosis, and phosphorus metabolic activities. In the cellular component group, most of the selected genes were in relationship with the extracellular matrix and cell surface. In the molecular function group, the selected genes were associated with the binding with identical protein, the activities of growth factor and cytokine, and the binding with receptors. In the KEGG signaling pathway group, the selected genes were related to the interaction between cytokines and their receptors, HIF-1 and TNF signaling pathways, hepatitis B, apoptosis, and cancer-related pathways.

**Table 2:** The top six pathways in GO and KEGG enrichment analyses of the 72 genes related to both autophagy and LDD

Category	Term	Count	*P* value
GOTERM_BP_FAT	GO:0042127~regulation of cell proliferation	46	2.12E−30
GOTERM_BP_FAT	GO:0010562~positive regulation of phosphorus metabolic process	40	1.70E−29
GOTERM_BP_FAT	GO:0045937~positive regulation of phosphate metabolic process	40	1.70E−29
GOTERM_BP_FAT	GO:0008219~cell death	48	1.79E−28
GOTERM_BP_FAT	GO:0042981~regulation of apoptotic process	43	1.85E−28
GOTERM_BP_FAT	GO:0010941~regulation of cell death	44	2.20E−28
GOTERM_CC_FAT	GO:0005615~extracellular space	37	5.19E−19
GOTERM_CC_FAT	GO:0044421~extracellular region part	44	2.75E−10
GOTERM_CC_FAT	GO:0005576~extracellular region	47	1.36E−09
GOTERM_CC_FAT	GO:0005578~proteinaceous extracellular matrix	14	1.01E−08
GOTERM_CC_FAT	GO:0009986~cell surface	18	5.52E−08
GOTERM_CC_FAT	GO:0031012~extracellular matrix	15	1.25E−07
GOTERM_MF_FAT	GO:0005125~cytokine activity	20	7.59E−20
GOTERM_MF_FAT	GO:0005102~receptor binding	36	1.05E−18
GOTERM_MF_FAT	GO:0005126~cytokine receptor binding	20	3.81E−18
GOTERM_MF_FAT	GO:0008083~growth factor activity	13	5.03E−12
GOTERM_MF_FAT	GO:0042802~identical protein binding	23	3.69E−08
GOTERM_MF_FAT	GO:0019838~growth factor binding	9	8.56E−08
KEGG_PATHWAY	hsa04060:Cytokine-cytokine receptor interaction	22	3.02E−16
KEGG_PATHWAY	hsa05200:Pathways in cancer	24	4.51E−14
KEGG_PATHWAY	hsa05161:Hepatitis B	16	8.20E−13
KEGG_PATHWAY	hsa04210:Apoptosis	12	3.07E−12
KEGG_PATHWAY	hsa04668:TNF signaling pathway	14	4.18E−12
KEGG_PATHWAY	hsa04066:HIF-1 signaling pathway	13	2.18E−11

*GO* Gene Ontology, *KEGG* Kyoto Encyclopedia of Genes and Genomes

### PPI network and module analysis

**Fig. 4:**
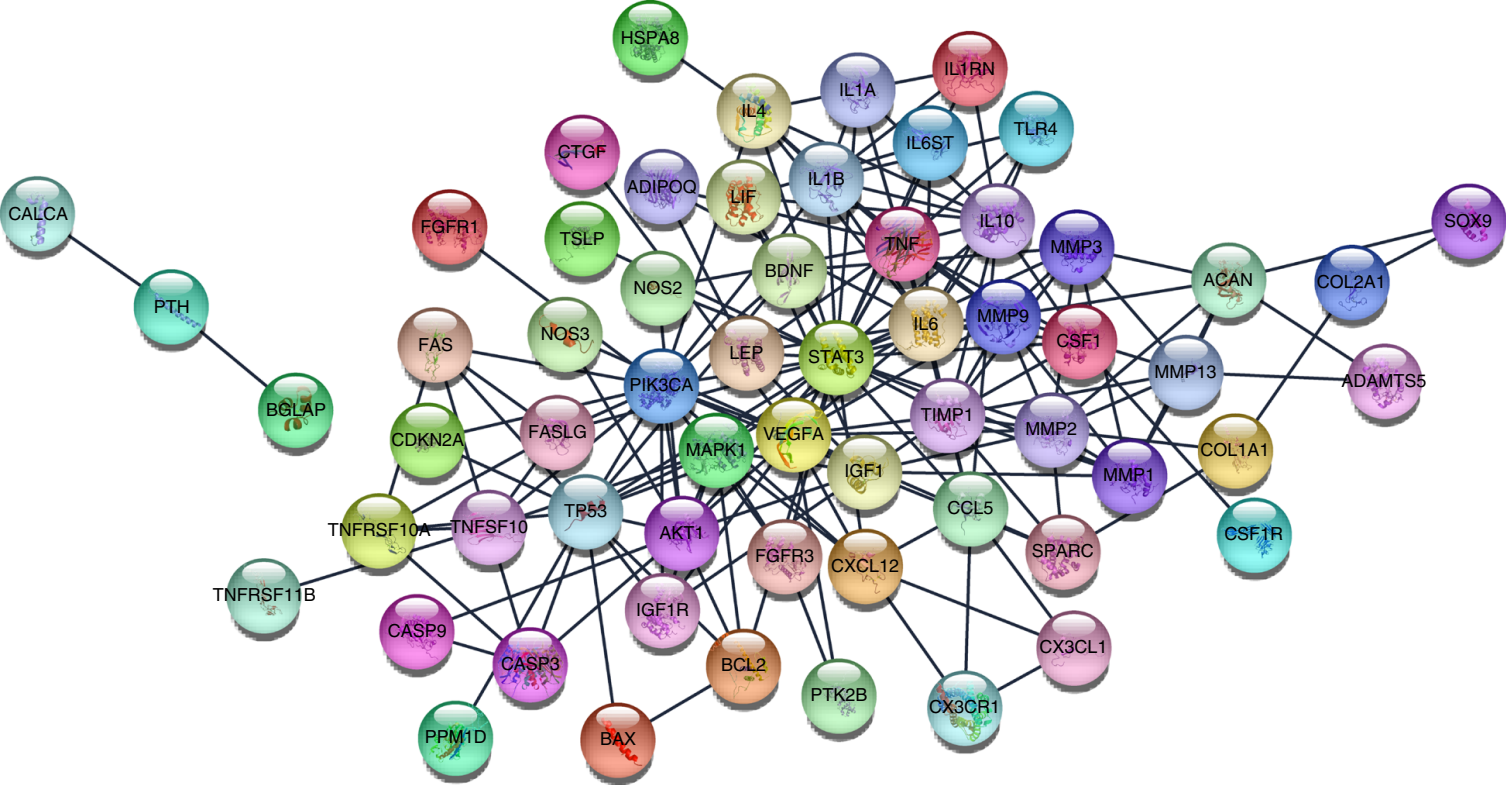
Based on the STRING online database, 70 genes were filtered into the PPI network

**Fig. 5:**
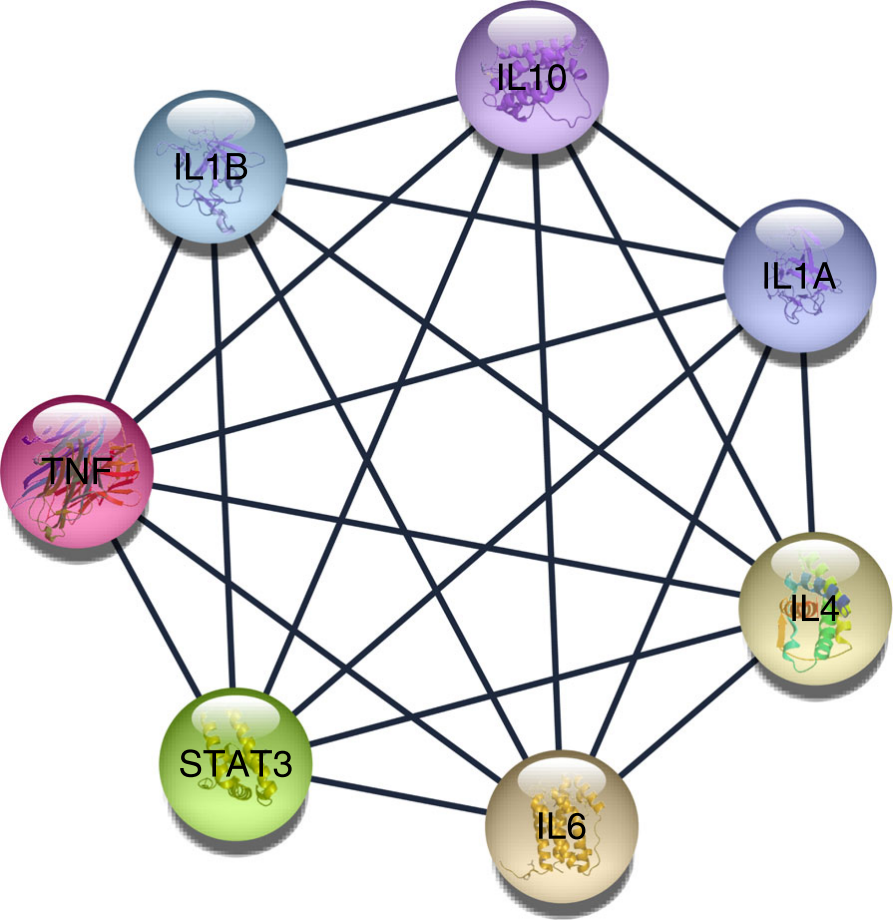
The most significant module 1 from the PPI network

**Fig. 6:**
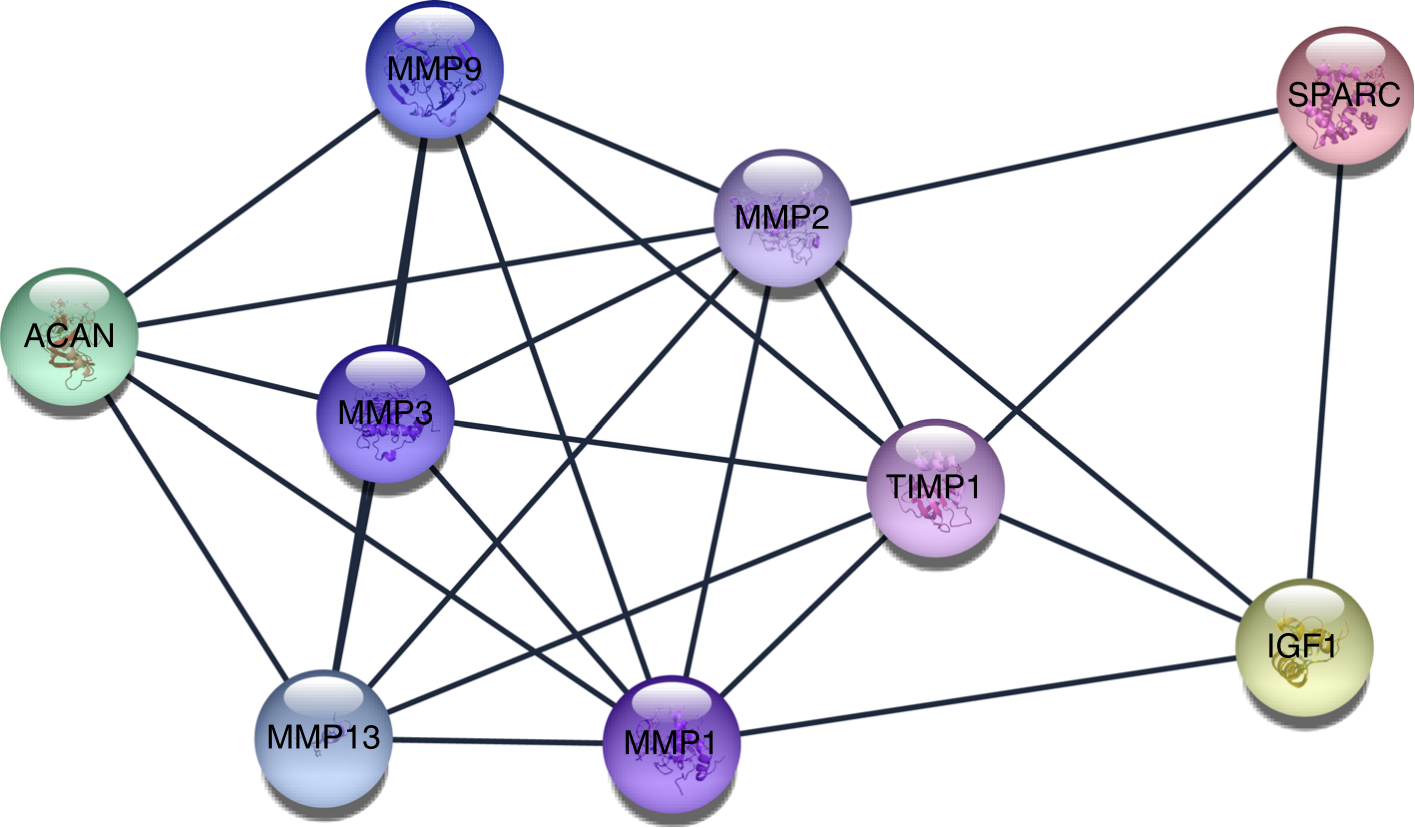
The second significant module 2 from the PPI network

**Fig. 7:**
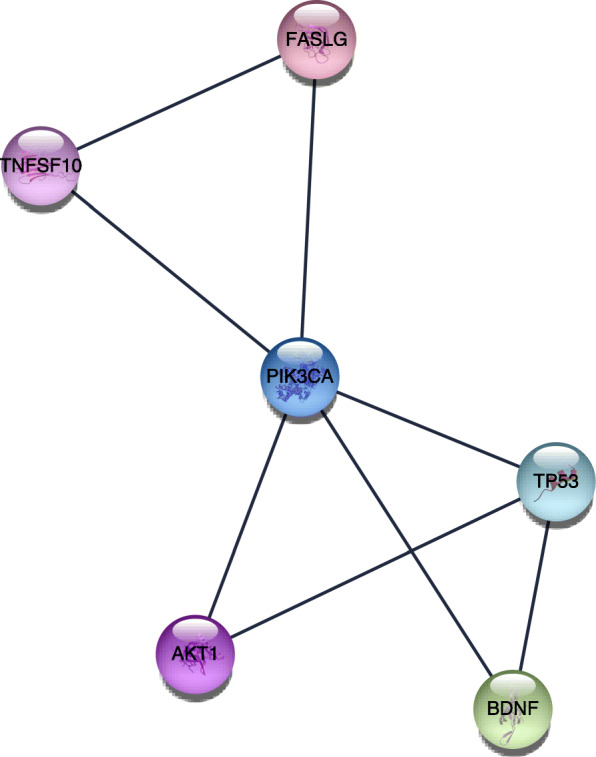
The third significant module 3 from the PPI network

The STRING website and Cytoscape software were used to analyze the 72 genes. There were 185 edges and 70 nodes/genes with scores >0.900 (highest confidence), and the PPI networks were established (Fig. 4). The MCODE plug-in was used, and 3 pivotal modules were selected. In module 1, there were seven nodes and twenty-one edges (Fig. 5), which were associated with the MAPK signaling pathway, the extracellular space, the binding with cytokine receptors, and the chemokine secretion (Table 3). In module 2, there were twenty-six edges and nine nodes (Fig. 6), which were related to the disassembly of the extracellular matrix, the proteins in the extracellular matrix, the activity of metalloendopeptidase, and the Estrogen signaling pathway (Table 4). In module 3, there were six nodes and eight edges (Fig. 7), which were related to the negative modulation of apoptosis, the complex of transferase, and the transportation of phosphorus-containing groups, MAPK signaling pathway, and the binding with cytokine receptors (Table 5).

**Table 3:** Functional and pathway enrichment of module 1 genes

Category	Term	Count	*P* value	Genes
GOTERM_BP_FAT	GO:1903426~regulation of reactive oxygen species biosynthetic process	6	6.31E−12	IL4, IL6, TNF, IL1B, IL10, STAT3
GOTERM_BP_FAT	GO:0032642~regulation of chemokine production	6	7.31E−12	IL4, IL6, TNF, IL1B, IL10, IL1A
GOTERM_BP_FAT	GO:0032602~chemokine production	6	1.04E−11	IL4, IL6, TNF, IL1B, IL10, IL1A
GOTERM_CC_FAT	GO:0005615~extracellular space	6	5.31E−05	IL4, IL6, TNF, IL1B, IL10, IL1A
GOTERM_CC_FAT	GO:0009897~external side of plasma membrane	3	0.004095871	IL4, IL6, TNF
GOTERM_CC_FAT	GO:0044421~extracellular region part	6	0.006314367	IL4, IL6, TNF, IL1B, IL10, IL1A
GOTERM_MF_FAT	GO:0005126~cytokine receptor binding	7	2.85E−11	IL4, IL6, TNF, IL1B, IL10, IL1A, STAT3
GOTERM_MF_FAT	GO:0005125~cytokine activity	6	3.44E−09	IL4, IL6, TNF, IL1B, IL10, IL1A
GOTERM_MF_FAT	GO:0070851~growth factor receptor binding	5	6.61E−08	IL4, IL6, IL1B, IL10, IL1A
KEGG_PATHWAY	hsa04630:Jak-STAT signaling pathway	4	1.75E−04	IL4, IL6, IL10, STAT3
KEGG_PATHWAY	hsa04668:TNF signaling pathway	3	0.00345185	IL6, TNF, IL1B
KEGG_PATHWAY	hsa04010:MAPK signaling pathway	3	0.01832436	TNF, IL1B, IL1A

**Table 4:** Functional and pathway enrichment of module 2 genes

Category	Term	Count	*P* value	Genes
GOTERM_BP_FAT	GO:0022617~extracellular matrix disassembly	7	5.09E−13	MMP9, ACAN, MMP3, MMP13, MMP2, MMP1, TIMP1
GOTERM_BP_FAT	GO:0030198~extracellular matrix organization	8	9.66E−12	MMP9, ACAN, SPARC, MMP3, MMP13, MMP2, MMP1, TIMP1
GOTERM_BP_FAT	GO:0043062~extracellular structure organization	8	9.86E−12	MMP9, ACAN, SPARC, MMP3, MMP13, MMP2, MMP1, TIMP1
GOTERM_CC_FAT	GO:0005578~proteinaceous extracellular matrix	8	4.33E−11	MMP9, ACAN, SPARC, MMP3, MMP13, MMP2, MMP1, TIMP1
GOTERM_CC_FAT	GO:0031012~extracellular matrix	8	6.42E−10	MMP9, ACAN, SPARC, MMP3, MMP13, MMP2, MMP1, TIMP1
GOTERM_CC_FAT	GO:0005615~extracellular space	7	2.24E−05	MMP9, IGF1, SPARC, MMP3, MMP13, MMP2, TIMP1
GOTERM_MF_FAT	GO:0004222~metalloendopeptidase activity	5	1.84E−07	MMP9, MMP3, MMP13, MMP2, MMP1
GOTERM_MF_FAT	GO:0008237~metallopeptidase activity	5	1.48E−06	MMP9, MMP3, MMP13, MMP2, MMP1
GOTERM_MF_FAT	GO:0004252~serine-type endopeptidase activity	5	4.78E−06	MMP9, MMP3, MMP13, MMP2, MMP1
KEGG_PATHWAY	hsa04066:HIF-1 signaling pathway	2	0.067876196	IGF1, TIMP1
KEGG_PATHWAY	hsa04915:Estrogen signaling pathway	2	0.069936289	MMP9, MMP2
KEGG_PATHWAY	hsa04668:TNF signaling pathway	2	0.075412069	MMP9, MMP3

**Table 5:** Functional and pathway enrichment of module 3 genes

Category	Term	Count	*P* value	Genes
GOTERM_BP_FAT	GO:1901214~regulation of neuron death	5	2.74E−07	AKT1, BDNF, TP53, PIK3CA, FASLG
GOTERM_BP_FAT	GO:0043066~negative regulation of apoptotic process	6	2.83E−07	AKT1, TNFSF10, BDNF, TP53, PIK3CA, FASLG
GOTERM_BP_FAT	GO:0043069~negative regulation of programmed cell death	6	3.02E−07	AKT1, TNFSF10, BDNF, TP53, PIK3CA, FASLG
GOTERM_CC_FAT	GO:0061695~transferase complex, transferring phosphorus-containing groups	2	0.084109658	TP53, PIK3CA
GOTERM_MF_FAT	GO:0005126~cytokine receptor binding	3	0.002992627	TNFSF10, BDNF, FASLG
GOTERM_MF_FAT	GO:0005102~receptor binding	4	0.007267044	TNFSF10, BDNF, TP53, FASLG
GOTERM_MF_FAT	GO:0051721~protein phosphatase 2A binding	2	0.0086928	AKT1, TP53
KEGG_PATHWAY	hsa04010:MAPK signaling pathway	4	4.65E−04	AKT1, BDNF, TP53, FASLG
KEGG_PATHWAY	hsa04151:PI3K-Akt signaling pathway	4	0.001159592	AKT1, TP53, PIK3CA, FASLG
KEGG_PATHWAY	hsa04150:mTOR signaling pathway	2	0.041464218	AKT1, PIK3CA

### The interactions between drugs and genes

Using three important modules, 22 genes were selected as possible drug targets, and 32 autophagy-related drugs were identified as potential agents used for LDD therapy (Table 6). Possible gene targets were as follows: *TP53* (two drugs), *IL-6* (one drug), *MMP1* (three drugs), *STAT3* (one drug), *MMP9* (two drugs), *TNF* (eleven drugs), *IL1B* (three drugs), *PIK3CA* (five drugs), *IL-1A* (one drug), *MMP* (two drugs), *AKT1* (three drugs), and *MMP13* (three drugs). These drugs were generally approved for treating osteoarthritis, psoriasis, vascular wrist joint disease, malignancy, idiopathic pulmonary fibrosis, and inflammatory diseases.

**Table 6:** Candidate drugs targeting genes

Number	Drug	Gene	Interaction type	Score	Approved?	Reference (PubMed ID)
1	Acitretin	*STAT3*	Inhibitor	1	Yes	None found
2	Adalimumab	*TNF*	Inhibitor	12	Yes	12044041
3	Arsenic trioxide	*AKT1*	Inducer	6	Yes	12472888
4	Aspirin	*TP53*	Acetylation	2	Yes	21475861
5	Bortezomib	*TP53*	Inhibitor	1	Yes	None found
6	Canakinumab	*IL1B*	Inhibitor	7	Yes	19169963
7	Candicidin	*PIK3CA*	Inhibitor	3	Yes	26839307
8	Captopril	*MMP2*, *MMP9*	Inhibitor	7	Yes	12381651
9	Certolizumab pegol	*TNF*	Inhibitor	6	Yes	22917017
10	Doxycycline calcium	*MMP1*, *MMP13*	Inhibitor	1	Yes	None found
11	Doxycycline hyclate	*MMP1*, *MMP13*	Inhibitor	1	Yes	None found
12	Doxycycline hydrate	*MMP1*, *MMP13*	Inhibitor	1	Yes	None found
13	Etanercept	*TNF*	Inhibitor	12	Yes	10375846
14	Everolimus	*AKT1*	Inhibitor	3	Yes	None found
15	Gallium nitrate	*IL1B*	Inhibitor	3	Yes	16122880
16	Glucosamine	*MMP9*	Antagonist	6	Yes	12405690
17	Golimumab	*TNF*	Inhibitor	6	Yes	21079302
18	Idelalisib	*PIK3CA*	Inhibitor	5	Yes	26466009
19	Inamrinone	*TNF*	Inhibitor	6	Yes	11805217
20	Infliximab	*TNF*	Inhibitor	17	Yes	16456024
21	Lenalidomide	*TNF*	Inhibitor	2	Yes	None found
22	Nelfinavir	*AKT1*	Inhibitor	1	Yes	None found
23	Oxazepam	*PIK3CA*	Inhibitor	1	Yes	None found
24	Pentoxifylline	*TNF*	Antibody	1	Yes	None found
25	Phenmetrazine	*PIK3CA*	Inhibitor	33	Yes	27672108
26	Pirfenidone	*TNF*	Inhibitor	1	Yes	None found
27	Pomalidomide	*TNF*	Inhibitor	2	Yes	22917017
28	Rilonacept	*IL1A*, *IL1B*	Binder	5	Yes	23319019
29	Siltuximab	*IL6*	Inhibitor	4	Yes	8823310
30	Thalidomide	*TNF*	Inhibitor	11	Yes	8755512
31	Tiludronic acid	*MMP2*	Inhibitor	1	Yes	None found
32	Yohimbine	*PIK3CA*	Inhibitor	35	Yes	27672108

## Discussion

The selected 22 genes and their targeted drugs and the related pathways were classified.

### Genes, targeted agents, and related pathways involved in LDD pathology

#### Genes, targeted agents, and gene-related pathways in association with disc catabolism

Matrix metalloproteinases (MMPs) are involved in the extracellular matrix proteins (ECM) degradation. Upregulated MMPs or decrease of their inhibitors (TIMPs) could result in an imbalance in ECM.

MMP-1: The expression of MMP-1 in LDD was elevated [17]. Doxycycline hydrate, doxycycline calcium, and doxycycline hyclate specifically inhibited MMP13 and MMP1.

MMP-2: MMP-2 might play an important role in the pathogenesis of LDD and could be a possible target in treatment [18]. Captopril and tiludronic acid are specific agents inhibiting MMP-2.

MMP-9: MMP-9 and IL-1α levels were increased in the degenerated lumbar disc as the disease progressed [19]. Glucosamine and captopril are specific agents inhibiting MMP-9.

MMP-13 and MMP-3: Estradiol could protect nucleus pulposus cells (NPC) from apoptosis caused by deprivation of the serum and regulate MMP-13 and MMP-3 levels via promoting autophagy [20]. In addition, it was found that BRD4 inhibited the expression of MMP-13 in diabetics-related degeneration of intervertebral disc via modulating autophagy, NF-κB, and MAPK pathways [21].

TIMP1: MMPs could be inhibited by the proteins encoded by the genes in the TIMP family. Kwon et al. presented that MMP-1, MMP-3, and IL-8 levels were remarkably elevated in lumbar disc cells under hypoxia condition, while TIMP-2 and TIMP-1 levels were downregulated [22].

#### Genes, targeted agents, and related pathways in disc anabolism

IGF1: Insulin-like growth factor 1 (IGF-1) and its receptor (IGF1R) can modulate the synthesis of ECM and play an important part in the normal function of the disc. It was reported that downregulated IGF1R could promote LDD in rats [23]. The PI3k/Akt signaling pathway was activated by IGF-1 to prevent LDD [24].

ACAN: The protein encoded by ACAN is an essential ingredient in the extracellular matrix of cartilage tissues. Metformin could increase the expression levels of anabolic genes like Col2a1 and Acan and suppress catabolic genes like Adamts5 and Mmp3 in NPC [25].

SPARC: As a kind of glycoprotein, SPARC plays a pivotal part in regulating the interaction between matrix and cells. Gruber et al. suggested that the expression of the SPARC gene in LDD decreased [26].

In summary, the above studies have shown that the long-term imbalance between catabolism and anabolism in the intervertebral disc changes its composition, contributing to LDD. Nonetheless, autophagy can modulate this imbalance, thereby inhibiting LDD.

### Genes, targeted agents, and pathways in relationship with inflammatory factors in LDD

IL1β: It is considered as an essential mediator in inflammatory responses. Zhang et al. presented that melatonin regulated the remodeling of extracellular matrix induced by IL-1β in human NPC and ameliorated inflammation and degeneration of intervertebral disc in rats [27]. Gallium nitrate and canakinumab are specific agents inhibiting L1β.

Tumor necrosis factor alpha (TNF-α): TNF-α encodes the pro-inflammatory cytokine belonging to the TNF family. It was found that TNF was a critical factor in LDD [28]. Furthermore, it was demonstrated that TNF-α could increase reactive oxygen species (ROS) production in cells and cause osteogenic differentiation and cellular senescence in cartilage endplate stem cells (CESCs); however, autophagy could protect CESCs from oxidative injuries elicited by TNF-α and senescence [29]. Thalidomide, pirfenidone, certolizumab pegol, lenalidomide, etanercept, infliximab, golimumab, inamrinone, and pomalidomide are specific agents inhibiting TNF. Additionally, pentoxifylline acts as a specific antibody for TNF.

IL-6: IL-6 plays a pivotal part in inflammatory responses and the maturation of B cells. It was found that microRNA-21 levels were elevated in lumbar intervertebral discs of patients with nerve root pain, which could enhance IL-6 mediated inflammation and attenuate autophagy [30]. Siltuximab is a specific agent inhibiting IL-6.

IL-1a: IL-1a cytokine could be secreted by macrophages and monocytes. Cellular injuries could promote the hydrolysis of the premature IL-1a cytokine and its mature form could induce apoptosis. Chen et al. presented that IL-1α participated in LDD pathogenesis via enhancing the enzymes related to the degradation of extracellular matrix and suppressing the production of extracellular matrix [31]. Rilonacept is a specific binder to IL-1β and IL-1A.

IL-10 and IL-4:shDNMT1 could reduce expressions of TNFα, IL-6, and IL-1β; increase expressions of IL-10 and IL-4; and ameliorate apoptosis in the degenerated discs and LDD-related pain [32]. Furthermore, Hanaei et al. showed that genetic alterations in anti-inflammatory genes could destroy intervertebral disc homeostasis and cause degeneration [33].

STAT3: STAT3 protein regulates the expression levels of multiple genes and thus has an essential part in various cellular activities including cell apoptosis and growth. Acitretin is a specific agent inhibiting STAT3.

To conclude, the inflammation-related factors accelerated disc degeneration via increasing the production of the enzymes related to extracellular matrix degradation, but autophagy could ameliorate inflammatory responses to protect the intervertebral disc.

### There are 4 significant autophagy-related pathways in LDD

#### Three autophagy-associated pathways participate in ameliorating neuro-inflammation and apoptosis via enhancing autophagy in lumbar disc degeneration

##### AMPK signaling pathway

It was reported that activating autophagy through the AMPK/mTOR pathway was a kind of cellular adaptation when the cells were injured by hyperosmotic stress [34]. Besides, resveratrol reduced MMP-3 levels induced by TNF-α in NPC via the activation of autophagy through the AMPK/SIRT1 signaling pathway [35]. Zhang et al. proved that naringin could promote the autophagy via the AMPK signaling pathway to attenuate apoptosis induced by oxidative stress in NPC [36].

##### mTOR signaling pathway

Jiang et al. showed that glucosamine could stimulate autophagy through the mTOR pathway and protect NPC after hydrogen peroxide (100 μM H_2_O_2_) or IL-1β treatment [37]. Autophagy and the mTOR signaling pathway were activated when cells in the intervertebral disc were of low nutrient, including low glucose, oxygen, or pH [38].

##### PI3K-Akt signaling pathway

Autophagy of NPC could be induced by compression stress via suppressing the PI3K/AKT/mTOR pathway and stimulating the JNK pathway [39]. Guo et al. demonstrated that resveratrol could promote the synthesis of the matrix in NPC via increasing autophagy through the PI3K/Akt pathway in oxidative stress (100 μM H_2_O_2_) [40]. Moracin M could repress inflammation in NPC through the PI3K/Akt/mTOR pathway [41].

To conclude, deprivation of nutrients, hyperosmotic condition, compression damage, inflammation factors, and oxidative injuries could enhance autophagy via various pathways. Afterwards, the activated autophagy could reduce apoptosis, decrease catabolism through downregulating MMPs, enhance the synthesis of the matrix in NPC, and ameliorate inflammation in NPC. *TNFSF10*, *AKT1*, and *PIK3CA* participated in these pathways.

AKT1: In neurological system development, AKT plays an essential part in mediating neuron survival induced by growth factors. Arsenic trioxide is a specific agent stimulating AKT1, while nelfinavir and everolimus have the opposite effects.

PIK3CA: PIK3CA protein represents catalytic subunit. Yohimbine, oxazepam, candicidin, phenmetrazine, and idelalisib are specific agents inhibiting PIK3CA.

TNFSF10: TNFSF10 protein belongs to the family of TNF ligands. Caspase 3, MAPK8/JNK, and caspase 8 were proved to be activated after TNFSF10 bound with its receptor.

#### There is an autophagy-associated pathway in LDD

##### ERK signaling pathway

BDNF, TP53, and FASLG play an important role in this pathway.

FASLG: FASLG belongs to the TNF family. FASLG protein could stimulate apoptosis after binding with FAS.

TP53: TP53 protein works as a tumor suppressor containing domains of oligomerization, transcriptional activation, and DNA binding. Bortezomib is a specific agent inhibiting TP53.

BDNF: BDNF protein belongs to the nerve growth factor family. The survival of neurons could be enhanced after BDNF protein is bound with its receptors in the brain of adults.

ERK signaling pathway was in a close relationship with LDD. Autophagy reduced NPC apoptosis induced by compression through the MEK/ERK/NRF1/Atg7 pathways [42]. Nonetheless, Chen et al. proved that H_2_O_2_ could stimulate autophagy in an early phase through the ERK/mTOR pathway, and the apoptosis rate of the cells injured by H_2_O_2_ (400 μM) could be decreased by inhibiting autophagy [43].

In summary, oxidative injuries (H_2_O_2_) could stimulate autophagy via various pathways. According to Chen et al.’s study, apoptosis could be promoted when autophagy was induced by 400-μM H_2_O_2_. In the research by Jiang and Gao, apoptosis could be inhibited when autophagy was induced by 100-μM H_2_O_2_. Based on previous research, it could be concluded that oxidative stress of various levels could result in autophagy which played a variety of roles.

As such, it could be speculated on the associations among apoptosis, necrosis, and autophagy. Autophagy has a bidirectional activity both in inhibiting and inducing apoptosis. However, from our point of view, apoptosis induced by autophagy might have benefits. For the cells which are severely injured, autophagy can promote programmed apoptosis so that greater damages induced by necrosis can be avoided, and thus, more energy can be reserved to repair the cells which have milder injuries. Generally, autophagy can be considered as a beneficial biological process.

There is also a limitation in our research. The function of the selected genes was not proved through experiments but obtained from databases. Therefore, it remains to be further verified by molecular biology experiments.

## Conclusion

To conclude, a method was proposed to discover possible key genes, signaling pathways, and potential drugs in relationship with autophagy and LDD. There were 22 possible genes, 4 pathways, and 32 potential drugs, providing a theoretical basis and new insight for basic research and treatment of LDD. Nonetheless, experiments are required in future research to verify the function of the selected genes, pathways, and drugs.

## Data Availability

All data are available upon request.

## Abbreviations

BP: Biological processes
LDD: Lumbar disc degeneration
KEGG: Kyoto Encyclopedia of Genes and Genomes
GO: Gene Ontology
CC: Cellular components
MCODE: Molecular complex detection
STRING: Search Tool for the Retrieval of Interacting Genes
MMPs: Matrix metalloproteinases
CESCs: Cartilage endplate stem cells
ECM: Extracellular matrix
IL-6: Interleukin-6
IGF-1: Insulin-like growth factor 1
TIMPs: Tissue inhibitors of metalloproteinases
TNF-α: Tumor necrosis factor alpha
NPC: Nucleus pulposus cells
ROS: Reactive oxygen species
PPI: Protein-protein interaction
DGIdb: Drug Gene Interaction Database
IL-1β: Interleukin-1β
MF: Molecular function
